# ω-3 and ω-6 Polyunsaturated Fatty Acids, Obesity and Cancer

**DOI:** 10.3390/nu12092751

**Published:** 2020-09-10

**Authors:** Stefania D’Angelo, Maria Letizia Motti, Rosaria Meccariello

**Affiliations:** Dipartimento di Scienze Motorie e del Benessere, Università di Napoli Parthenope, via Medina 40, 80133 Napoli, Italy; stefania.dangelo@uniparthenope.it (S.D.); motti@uniparthenope.it (M.L.M.)

**Keywords:** obesity, ω-3PUFAs, ω-6PUFAs, endocannabinoids, CRC, fatty acids

## Abstract

Recently, nutraceutical bioactive compounds in foods have been discovered for their potential health benefits regarding the prevention of chronic disorders, such as cancer, and inflammatory, cardiovascular, and metabolic diseases. Dietary omega-3 polyunsaturated fatty acids (ω-3PUFAs), including alpha-linolenic acid, docosapentaenoic acid, and eicosapentaenoic acid, are mostly attractive. They are available for the customers worldwide from commonly used foods and/or as components of commercial food supplements. The anti-inflammatory and hypotriglyceridemic effects of these fatty acids are well known, whereas pro-inflammatory properties have been recognized in their dietary counterparts, the ω-6PUFAs. Both ω-3 and ω-6PUFAs contribute to the production of lipid mediators such as endocannabinoids that are notably involved in control of food intake, energy sensing, and food–related disorders. In this review, we present ω-3 and ω-6PUFAs and their derivatives, endocannabinoids; discuss the anti-obesity effects of ω-3PUFAs; their roles in inflammation and colorectal cancer development; and how their action can be co-preventative and co-therapeutic.

## 1. Introduction

The prevalence of obesity has increased worldwide. Obesity represents a major health challenge because it substantially increases the risk of comorbidities, including cardiovascular disease, hypertension, type 2 diabetes, dyslipidemia, nonalcoholic fatty liver disease, obstructive sleep apnea, and some cancers, thereby contributing to a decline in both quality of life and life expectancy [1]. This syndrome is a complex condition involving social, biological, and psychosocial factors. The genesis of obesity is multifactorial: it is characterized by chronic low-grade inflammation, primarily due to an imbalance between the production/secretion of pro-inflammatory cytokines vs. anti-inflammatory cytokines [2]; deregulated lipid and glucose metabolism in metabolic organs is thought to be a critical factor. High-calorie diets and sedentary lifestyles are the most important factors in the development of obesity. As a consequence, global anti-obesity strategies focus on dietary and lifestyle modifications. In fact, weight loss, energy restriction, and nutrient dense diets can restore this imbalance, at least in part [3]. Therefore, the most used approaches aim at suppressing appetite, normalizing lipid metabolism, and increasing energy expenditure [4], through limitation of sugar and fat consumption, promotion of physical activity, consumption of fruits and vegetables, and pharmacological approaches.

Dietary interventions using natural bioactive food compounds have emerged as promising therapeutic tools for metabolic diseases, with limited deleterious side effects. Composition of the diet may affect metabolic and endocrine functions and overall energy balance [5]. Studies conducted in both animal models and humans support the assertion that dietary bioactive compounds can increase energy expenditure and thermogenesis, providing benefits in preventing/limiting obesity. Most health recommendations emphasize diets rich in fruits and vegetables, which have lower caloric density and higher nutrient density [6]. Such diets would provide significant amounts of phytochemicals, bioactive components with nutraceutical effects due in part to their anti-oxidant and anti-inflammatory properties [7]. Natural bioactive compounds, for example, the polyphenols (epigallocatechin, resveratrol, curcumin, quercetin, oleuropein, anthocyanins, ellagic acid, and others), have been studied as factors with possible indirect or direct impacts on specific molecular pathways, associated with the pathophysiologies of different syndromes [8,9] due to their well-documented anti-oxidant [10,11,12], anti-proliferative [13,14,15,16], anti-inflammatory, anti-cancer, anti-aging, and anti-obesity effects [17,18,19,20,21]. In addition to polyphenols, other nutraceuticals with an anti-obesity effects are the dietary ω-3 polyunsaturated fatty acids (PUFAs), which can act on adipose tissue inflammation, in contrast to omega-6 (ω-6) PUFAs, which exhibit pro-inflammatory properties. Both ω-3 and ω-6PUFAs contribute to the production of lipid mediators such as endocannabinoids, which are notably involved in control of food intake, energy sensing, and food–related disorders; reproduction; inflammation; the stress response; and cancer, among other things [22,23,24,25,26,27,28].

Therefore, in this review article we present ω-3 and ω-6PUFAs and their derivatives, endocannabinoids; discuss the anti-obesity effects of ω-3PUFAs; their role in inflammation and colorectal cancer (CRC) development; and co-preventative and co-therapeutic applications.

## 2. ω-3. and ω-6PUFAs

The amount and type of dietary fat in the diet are important factors influencing adipose tissue function and whole-body metabolism, with important health ramifications. Fatty acids (FA) are hydrocarbon chains with a carboxyl group at one end and a methyl group at the other. FA species are classified by their varying degrees of saturation into three main classes: saturated fatty acids (SFA), monounsaturated fatty acids (MUFA), and polyunsaturated fatty acids (PUFA) [29].

SFAs have a simple carbon chain containing no double bonds; MUFAs contain one double bond; and PUFAs are classified as carbon chains containing two or more double bonds. The variations in the chemical structures of these diverse classes can lead to dissimilar physiological activities. For example, SFA has been linked to the development of metabolic dysfunction; contrariwise, MUFAs and PUFAs have helpful activities in metabolism [30].

PUFAs are additionally classified into ω-3 and ω-6 groups, based on the position of the first double bond from the methyl end of the fatty acid. The structural dissimilarities of these FAs also give rise to functional differences, in terms of their actions on inflammation and metabolism [5].

ω-3 FAs are PUFAs with more than one carbon-carbon double bond in their backbones. They are polyunsaturated because their chains have numerous double bonds. One way in which a FA is named derives from the position of the first double bond, counted from the tail, that is, the omega (ω-) or the n-end. Thus, in ω-3 FAs, the first double bond is between the third and fourth carbon atoms from the tail end; then, they have a double bond at the third carbon from the methyl end of the carbon chain.

Humans do not own the essential ω-3 desaturase to add a double bond at the 15th carbon of a long chain FAs, and are, therefore, unable to endogenously synthesize α-linoleic acid (ALA 18:3n-3) and linoleic acid (LA 18:2n-6), making them vital FAs.

In addition to ALA, ω-3 PUFAs can be defined as a heterogeneous mix of FAs, among which eicosapentaenoic acid (EPA, 20:5n-3) and docosapentaenoic acid (DHA, 22:6n-3) are presently thought to be the most bioactive of the ω-3 species; however, docosapentaenoic acid (DPA, 22:5), an intermediate of EPA and DHA, may also have positive health properties [30,31].

ω-6PUFAs are also essential fatty acids and normally have metabolically distinct properties to ω-3PUFAs. While the human body cannot synthesize ω-3 and ω-6PUFAs, it does have the capability to further metabolize these FAs through stages of elongation and desaturation. ALA can be metabolized to EPA and DHA by Δ6 desaturase and Δ5 desaturase correspondingly, while LA is transformed to arachidonic acid (AA 22:4n-6). However, the change of ALA to DHA is very inefficient with <10% transformation in females and <3% in males [30,32]. While ALA is the favorite substrate for Δ6 desaturase, plenty of dietary linoleic acid has been observed to suppress the change of ALA to DHA, which may be a confounding factor in these data. Evidence suggests that supplementing with stearidonic acid (18:3n-3) may increase the efficiency of transformation to DHA, demonstrating Δ6 desaturase as a rate limiting step. There is also a degree of individual change in the lipidome following ω-3 integration in humans, which may be a factor in the equivocal metabolic conversion measured in many human integration trials [30,33].

The structural differences of the FAs give rise to functional dissimilarities, in terms of their actions on inflammation and metabolism. For example, due to the pro-inflammatory actions of saturated FAs, intake of these molecules is associated with an increase in cardiovascular disease risk. In contrast, ω-3PUFAs have anti-inflammatory capability, and their intake is connected to a decrease of in cardiovascular disease risk. Dietary FAs are involved in glucose-insulin homeostasis and modulating adipose tissue properties [5]. High saturated-fat intake causes adipose tissue inflammation and obesity in mice; those effects can be partially reduced when these high-fat diets are energy-restricted [29]. Instead, dietary EPA supplementation ameliorates adipose tissue inflammation, regardless of adipose tissue mass. These data, taken as a whole, demonstrate the importance of AF in modulating the adipose tissue properties [34].

Saturated FA, generally, contributes to adipose tissue inflammation, probably due to TLR-2 and TLR-4 activation, and switching of downstream pro-inflammatory signaling pathways comprising the nuclear factor kappa-light-chain-enhancer of activated B cells (NF-κB) pathway [29]. In contrast, ω-3 FA, primarily DHA and EPA, mitigate adipose tissue inflammation in diverse animal models of obesity [34]. Compared to ω-6PUFAs such as AA, ω-3PUFAs generate less eicosanoids with inflammatory properties. Additionally, ω-3PUFAs competitively decrease AA-mediated inflammatory eicosanoid prostaglandin E2 (PGE2) synthesis. As a whole, food sources rich in healthy fats can provide health benefits in both the long and short term.

**Figure 1:**
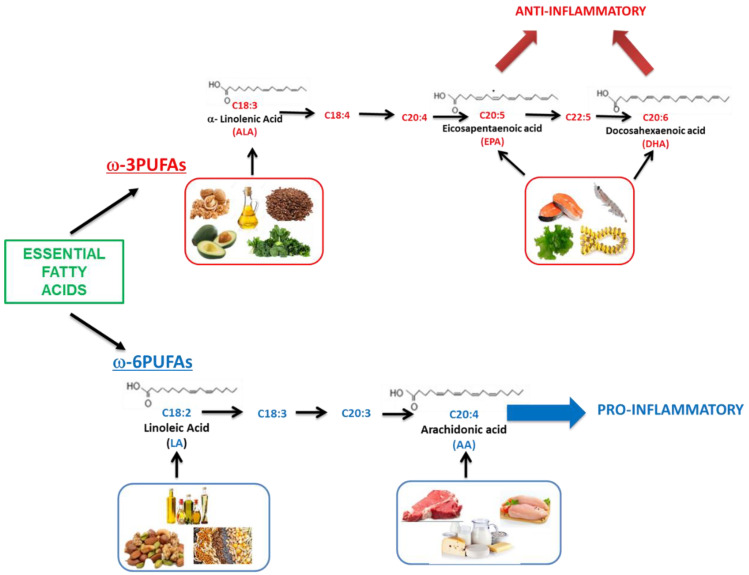
Essential fatty acids and dietary source (AA: arachidonic acid; ALA: α-linolenic acid; DHA: docosahexaenoic acid; EPA: eicosapentaenoic acid; LA: linoleic acid; PUFA: polyunsaturated fatty acid).

Figure 1 summarizes the essential FAs and their dietary sources. In general, the primary source of ω-3PUFAs in the human diet is marine products, in particular phytoplankton that enters at multiple levels in the food chain [34], fatty fish, and cod liver oil, food rich in DHA and EPA [35,36].

In plants, ALA can be extracted from seeds such as flaxseed (linseed), green leafy vegetables, legumes, and nuts. Vegetable oils such as sunflower, corn, perilla, canola, and soybean are the principal sources of LA, and they provide a small amount of ALA [31].

### 2.1. Endocannabinoids: PUFAs Derivatives Involved in the Central and Peripheral Control of Food Intake

The endocannabinoid system (ECS) comprises lipid mediators capable of binding to and activating cannabinoid receptors—traditionally, the central and the peripheral cannabinoid receptor, CB1 and CB2 respectively, membrane G-coupled receptors originally found to be mainly expressed in the brain and peripheral tissues. The system also includes a large set of biosynthetic and metabolizing enzymes and transporters [37]. Since the discovery in the 1990s of the first endocannabinoids, anandamide (AEA) and 2-arachinonoylglycerol (2-AG), the relevance of ECS has been progressively widening due to the inclusion in the system of non-canonical cannabinoid receptors (i.e., the transient receptor potential cation channel subfamily V member 1 (TRPV1); the orphan G-coupled receptors GPR18, GPR119, and GPR55; and also peroxisome proliferator-activated receptors (PPARs) such as PPARα and γ), the discovery of several “endocannabinoid-like” compounds, and the large spectrum of biological functions [38]. Currently ECS represents a conserved, widely-expressed signaling system involved in the control of most biological activities within the brain and peripheral tissues, from food intake to reproduction, the immune response, and cancer, among others [22,23,24,25,26,27,28,39], and it is susceptible to epigenetic modulation by diet [40].

**Figure 2:**
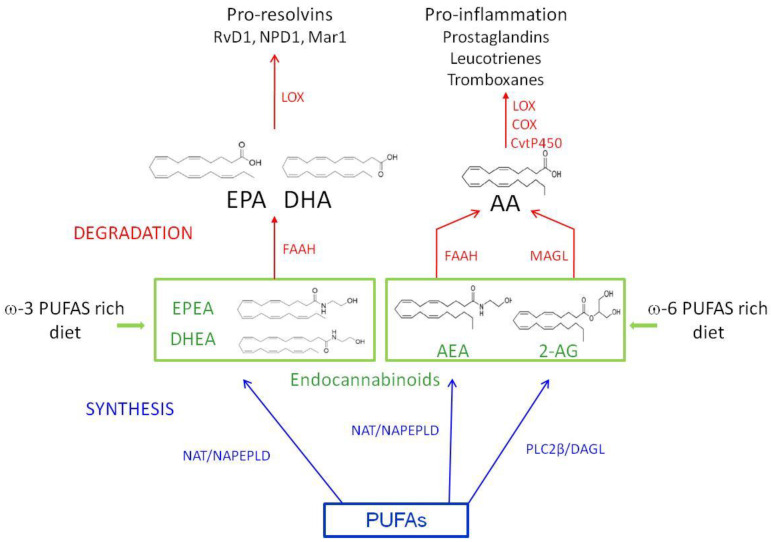
A schematic representation of the metabolic/hydrolyzing pathways linking ω-3 and ω-6, endocannabinoids, and inflammatory mediators. The syntheses of DHEA and EPEA, and AEA from ω-3 and ω-6 PUFAs respectively, requires the activity of N-acetyltransferase (NAT) followed by N-acyl phosphatidylethanolamine-specific phospholipase D (NAPEPLD). The synthesis of 2-AG from ω-6 PUFAs requires the subsequent activity of phospholipase Cβ (PLCâ) and diacylglycerol lipase (DAGL). The hydrolysis of the endocannabinoids requires the activity of the fatty acid amide hydrolase (FAAH) and monoacylglycerol lipase (MAGL). The activity of lipoxygenases (LOX), cyclooxygenase (COX), and cytochrome P450 enzymes (CyP450) drives the production of inflammation mediators. AA: arachidonic acid; AEA: anandamide; 2-AG: 2-arachinonoylglycerol; DHA: docosahexaenoic acid; DHEA: N-docosahexaenoyl ethanolamine; EPA: eicosapentaenoic acid; EPEA: N-eicosapentanoyl ethanolamine; Mar1: maresin 1; NPD1: neuroprotectin D1; PUFA: polyunsaturated fatty acid; RvD1: resolvin D1.

Traditionally, AEA and 2-AG are the N-ethanolamide and the glyceryl ester of ω-6PUFA AA respectively, and represent the main endogenous ligands of CB1 and CB2, with AEA having low CB2 affinity, and 2-AG capable of binding both receptors [41]. The endocannabinoids N-docosahexaenoyl ethanolamine (DHEA) and N-eicosapentanoyl ethanolamine (EPEA) are ω-3 DHA and ω-3 EPA derivatives respectively; docosahexaenoylglycerol (DHG) and eicosapentaenoyl glycerol (EPG) are the glycerol esters of ω-3 DHA and EPA derivatives and have been discovered following the investigations on 2-AG analogues. While ω-6 AA derivatives AEA and 2-AG bind the canonical receptors [41], ω-3 EPA/DHA derived endocannabinoids, and endocannabinoid-like compounds exhibit lower affinity binding to CB1/CB2 than AEA/2-AG, and in some cases bind the aforementioned non canonical cannabinoid receptors [42]. Currently, ω-3 derived endocannabinoids are known at lesser extent than canonical ones, and their basic characterizations support possible involvement in inflammation, neuroprotection, and cancer [42]. Details concerning the metabolic/hydrolyzing pathways linking ω-3 and ω-6, endocannabinoids, and inflammatory mediators are summarized in Figure 2.

Endocannabinoids, via CB1, have a recognized role as orexigenic factors, in that they stimulate food intake and body fat deposition [26,43]; at the periphery ECS activity is largely reported in the gastrointestinal tract, and liver and adipose tissue, along with possible involvement in the microbiota–gut–brain axis and obesity onset, as excellently reviewed in [23]. Obese subjects display high endocannabinoid tone in the plasma and brain; furthermore, altered expression of CB1 and higher endocannabinoid levels in the muscle, adipose tissue, pancreas, and liver have been reported ([23] for a recent review). Consistently, CB1 activation increases food intake [44], whereas its pharmacological and genetic impairment reduces food assumption, protecting against the development of obesity, liver steatosis, and related inflammation [45,46]. Traditionally endocannabinoid signaling via CB1 is involved in the central control of food intake exerting its activity within the hypothalamus. This brain region catches and integrates the environmental cues, including fuel availability, in order to maintain energy homeostasis [47], projects neuronal networks towards different nuclei within the hypothalamus or in the brain stem controlling both the homeostatic regulation of energy balance, and biological functions deeply related to energy homeostasis and availability, such as reproduction [26]. Appetite inhibiting neuropeptides, such as proopiomelanocortin (POMC) precursor and cocaine-amphetamine regulated transcript (CART), and appetite stimulating neuropeptides such as neuropeptide Y (NPY), melanin-concentrating hormone (MCH), and agouti related protein (AgRP), are produced within the hypothalamic arcuate nucleus (ARC) to mediate the adaptive changes in food intake and energy expenditure in response to nutrient availability and peripherally produced “metabolic sensors” (i.e., glucose from liver, interleukin-6 (IL-6) from muscle, leptin from white adipose tissue, insulin, amylin and pancreatic polypeptide from pancreas, glucagon-like peptide-1, gherlin, cholecystokinin and peptide YY from the gastro-intestinal tract, and lastly gut microbiome-derived signals) [23,26,47]. ECS activity may be affected by metabolic sensors and may modulate neuronal population producing orexigenic/anorexigenic-peptides [26]. Among “metabolic sensors,” leptin is the major peripheral indicators of body metabolic reserves acting as satiety signal [48]. In the natural mutant mice for *Leptin* gene, the Ob/Ob mice, over activation of ECS has been reported in the hypothalamus whereas leptin inhibits the hypothalamic activity of ECS [49]. Additionally leptin resistance, a condition causing food intake alteration and consequent obesity development, has been linked to the over-activation of the ECS [49], with a sex-specific epigenetic modulation of *cnr1*, the gene encoding CB1, following maternal high fat diet (HFD) [50]. Therefore, CB1 antagonist-based therapy has been used for the treatment of obesity, but, in spite the promising anti-obesity effects, the treatment caused severe psychiatric side effects and has been discontinued in patients [51].

A deep interplay between the synthesis and actions of the AA, DHA, and EPA-derived endocannabinoids and ECS occurred, as recently reviewed [52]. In this respect, many authors suggest that dietary PUFAs and in particular the ω-6/ω-3 ratio may affect the endogenous tone of endocannabinoids with consequences on health [26,53]. For example, in animal models fed for three or four months with a ω-3 deficient diet, low DHA levels in the brain with effects on ECS and synaptic plasticity have been discovered [54,55]; similarly, long-term EPA and DHA supplementation reduces AEA and 2-AG levels, with reciprocal increases in levels of the corresponding endocannabinoid-like EPA- and DHA-derived molecules [52].

Lastly, dietary intervention may epigenetically affect ECS in animal models, and cell lines [40]; and at present, a recurrent process potentially influencing the development of eating disorders such as binge-eating has been related to the epigenetic modulation of FAAH [56], the gene encoding for the main endocannabinoid hydrolyzing enzyme.

## 3. The Antiobesity Effects of ω-PUFAs

DHA and EPA exert numerous beneficial effects, and they act as natural hypolipidemics, decrease risk of cardiovascular syndromes and could prevent the progress of insulin resistance and obesity [57].

Human dietary intervention trials suggest that fish oil (EPA and DHA) supplementation might decrease waist circumference. ω-3PUFAs decrease adiposity by numerous effects. For example, DHA and EPA start AMP-activated protein kinase (AMPK), which in turn actives FA β-oxidation in adipose tissue [5]. DHA and EPA are also promoting mitochondrial biogenesis, which can increase energy metabolism [58].

In rodents, DHA and EPA also raise FA oxidation in the small intestine and liver in in vivo experiments. DHA and EPA prevent hepatic lipogenesis in an AMPK and PPARα-dependent manner [5]. PPARs are transcription factors that form heterodimers with retinoid X receptors in the promoter regions of different genes implicated in glucose and lipid e metabolism [59]. PPARγ works as a main regulator of adipogenesis and checks numerous genes and adipokines in glucose and lipid metabolism. ω-3PUFAs work as ligands for PPARγ, and it has been observed that PPARγ plays a evident role in the capability of ω-3PUFAs, particularly DHA, to prompt M2 macrophage polarization and thereby decrease inflammation since these data are not detected in PPARγ-knockdown RAW264.7 cells [60]. The EPA- and DHA-mediated increment in FA oxidation and decrease in lipogenesis could be accountable for their anti-obesity actions [61].

**Figure 3:**
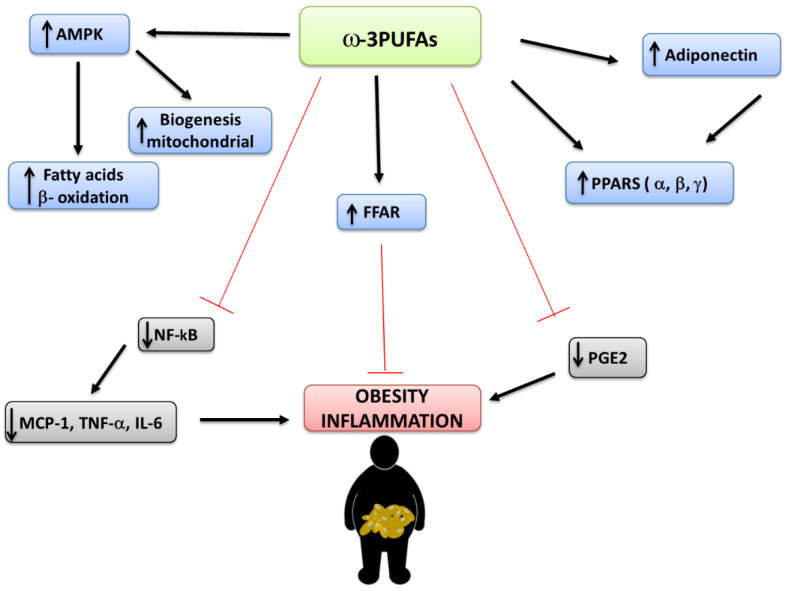
Some signaling mechanisms mediating effects of ω-3PUFAs (AMPK: AMP-activated protein kinase; FFAR: free fatty acid receptor; IL-: Interleukin-; MCP-1: Monocyte chemo attractant protein 1; NF-kB: nuclear factor-κB; PGE2: Prostaglandin E2; PPAR: Peroxisome proliferator-activated receptor; PUFA: polyunsaturated fatty acid; TNF-α: Tumor necrosis factor-alpha).

In Figure 3, some signaling mechanism-mediating effects of ω-3PUFAs are summarized.

DHA and EPA may act as anti-inflammatory agents directly. EPA improves adipose tissue inflammation and decreases insulin resistance. The ameliorations in adipokines’ profiles are characterized by rises in anti-inflammatory adipokines, such as adiponectin, and reductions in pro-inflammatory cytokines, such as IL-6, tumor necrosis factor-alpha (TNF-α), monocyte chemo attractant protein 1 (MCP-1), and plasminogen activator inhibitor 1 (PAI-1). EPA and DHA’s action of normalizing plasma adiponectin concentrations appears to be largely responsible for their insulin sensitizing action. This favorable activity on adiponectin secretion seems to be PPARγ-dependent, because fish oil fails to raise plasma adiponectin in PPARγ-null mice [62].

Prostaglandins, eicosanoids with pro-inflammatory action, are secreted by adipocytes. AA-originated eicosanoids such as thromboxane A2 and PGE2 possess stronger inflammatory action than EPA-originated ones. Since EPA contends with AA for incorporation into cell membranes, it is possible that enhancement dietary EPA intake decreases synthesis of AA-originated eicosanoids. Indeed, EPA hinders AA-induced secretion of PGE2 from 3T3-L1 adipocytes in vitro [63].

A close link was observed between inflammatory markers, BMI, and body fat percentage. NF-κB, a key transcription factor in gene expression and cytokine inflammation is inhibited by ω-3 PUFAs. Studies in humans and in vitro have shown that ω-3PUFAs are involved in the reduction of cytokines, such as IL-1, IL-6, and TNF-α, whose concentrations are high in cases of obesity [64]. The ω-3PUFAs behave as agonists for numerous free fatty acid receptors (FFARs) typical of different cell types, involved in both the inflammatory response and energy homeostasis. Some unsaturated and saturated long-chain fatty acids can activate FFAR1 and FFAR4 [65]. For example, FFAR4 stimulation prevents lipopolysaccharide (LPS)-mediated release of inflammatory cytokines, such as TNF-α and IL-6 in the macrophage-type RAW264.7 cell line [64].

Current data show that reduction of inflammation is an active process. EPA and DHA-derived resolvins and protectins are key examples of inflammation resolution agonists. Experiments involving treatment with resolvins or transgenic restoration of protectins have shown a slowdown of the adipose tissue macrophage infiltration, and enhanced insulin resistance in rodents. Secretion of these mediators could be another mechanism by which DHA and EPA ameliorate the inflammation in adipose tissue [5].

Human clinic trials have been organized to evaluate the effects of the intake of ω-3PUFAs (using as food different types of fish with different contents of DHA and EPA) on the variation of composition and body weight, and also on the evaluation of the caloric content of food intake. Fish oil and fishes have also been used in dietetic interventions of different duration and with or without associated physical activity, to evaluate a possible weight loss.

Participant-reported diet diaries show evident decreases in fat, carbohydrate, and total caloric intake with ω-3PUFAs integration [66], but others reported no variation in energy intake [29,67]. Since most trials only indicated total caloric intake, the action of ω-3PUFAs integration on macronutrient and energy intake should be repeated in larger trials to conclusively establish the action of these PUFAs on weight reduction in humans. Weight reduction data appear more encouraging when ω-3PUFA integration and calorie restriction are combined, but it is problematic to draw deductions due to the diversity of calorie limit programs in dissimilar trials. Combined ω-3PUFAs supplementation and calorie restriction compared to calorie restriction alone or replacement of saturated fatty acids determined a major amelioration in insulin resistance and reduction of TGs [68,69]. ω-3PUFAs could decrease body weight, thereby improving the metabolic profile through various mechanisms: alteration in adipokines release; modification of gene expression in adipose tissue; adipokine-mediated or adipokine-connected pathways; variation in carbohydrate metabolism; appetite suppression; rise in fat oxidation; intensification in energy expenditure (probably by thermogenesis); initiation of mechanisms related to muscle anabolism; and, lastly, epigenetic actions.

The adipose tissue increase in obesity happens via hyperplasia (augmentation in adipocyte number due to adipogenesis) and adipocyte hypertrophy (growth of adipocytes). Both ω-3 and ω-6 PUFAs can bind and/or control transcriptional factors that regulate genes implicated in pre-adipocyte differentiation. Principally, AA and its derivatives act as ligands for PPARγ and PPARδ to cause fat cell differentiation and quicken maturation by increasing lipoprotein lipase expression in vitro [70]. Importantly, concentrations of ω-6 and ω-3PUFAs in human subcutaneous tissue are associated with less adipocyte size; improved saturated FA concentrations lead to amplified fat cell size. Considering all these data, it is possible to propose that ω-3PUFAs stimulate adipogenesis and a healthy expansion of adipose tissue during positive energy balance, stimulating a metabolically healthy phenotype [70].

Numerous trials have observed that ω-3PUFAs modulate adipokine secretion. Obese individuals have high plasma leptin values indicative of leptin resistance. Weight-loss-connected to decrease in leptin could act on hunger and a lower metabolic rate and ultimately lead to weight regaining [71]. EPA integration reduces the decrease in blood leptin values, which happens during weight loss in obese women, proposing a potentially prominent role of EPA in weight loss conservation [72]. ω-3PUFA-mediated consequences on leptin are related to a various factors, such as energy balance and kind of diet, which could determine incompatible data anyway [29]. It has been suggested that the anti-inflammatory capacities of ω-3PUFAs integration cause a rise in adipocyte adiponectin synthesis and get better leptin sensitivity. This interaction could have a substantial influence on body weight control.

A study described evident sensations of fullness in the attendees who took higher ω-3 PUFA content food compared to those who took lower ω-3PUFA content meals both immediately and 2 h after eating the meal. [73]. Consequently, it is possible that an increase in the feeling of satiety after a meal rich in ω-3PUFA content can help weight loss by reducing the next food intake. Additionally, FFAR4 could mediate appetite reduction. ω-3PUFAs are agonists for FFAR4, which provokes the secretion of cholecystokinin, a hormone that is synthesized, is freed from the small intestine, and is related to appetite suppression [74].

Brown adipose tissue (BAT) is a particular fat that disperses excess energy into heat (non- shivering thermogenesis) through mitochondrial uncoupling protein 1 (UCP1). Current studies confirm the metabolic activity of BAT by revealing BAT as a crucial regulator in ensuring energy balance by rising thermogenic energy consumption. An important quantity of BAT is dose in healthy adults and most children and adolescents, but not in the obese adults, indicating that loss of operative BAT depots is a contributing obesity factor. Cold- and diet-induced thermogenesis mediated by UCPs in the presence of ω-3PUFA have been analyzed in some studies [75]. UCPs are inner mitochondrial proteins moving hydrogen ions across the mitochondrial inner membrane [76]. ω-3PUFAs enhance mitochondrial oxidative capability in skeletal muscle and WAT, probably through UCP-3 up-regulation, but not in liver or BAT. Nevertheless, because most trials were carried out at 20 °C, it is uncertain whether increment in mitochondrial oxidative capability is ω-3PUFA-mediated or cold caused. Mechanisms relating the role of ω-3PUFAs in probable induction of energy expense and reduction of body fat should be investigated further at different temperatures since thermogenic markers act even at 22 °C [76].

Control of lipid metabolism may change by ω-3PUFA type, and by fat store. For example, EPA is preferably aimed to β-oxidation, while DHA and DPA avoid catabolism and are stored in tissues. Furthermore, hormone-sensitive lipase, and gene expressions of fatty acid synthase, phosphoenol-pyruvate carboxykinase, and lipoprotein lipase, in retroperitoneal fat, are reduced with DHA and mixed EPA/DHA integration but not with EPA integration alone [77]. Additionally, ultimately, ω-3 PUFAs control lipid metabolism, encouraging fatty acid oxidation and repression of lipogenesis, causing a positive lipid profile and adipocyte metabolism.

The determination of ω-6 and ω-3PUFAs composition in the cell membrane of red blood cells (RBC) represents a biomarker of dietary intake and endogenous metabolism; in addition, it is a precise way to perform estimated studies and clinical trials in order to value their effects in weight increase and obesity. Harris et al. led a prospective study to observe the link between baseline RBC membrane phospholipids of ω-3PUFAs, ω-6PUFAs, ω-6/ω-3 ratio, and *trans* FA with the variations in body weight and the risk of becoming obese or overweight during a mean of 10.5 years follow up in the NIH Women’s Health Initiative Study. This prospective analysis provided a strongly suggestive sign that ω-3PUFAs in RBC membrane phospholipids are reversely connected, while *cis* ω-6, ω-6/ω-3 ratio and *trans* fatty acids are favorably related with weight gain [78].

### Recommendations for PUFAs Intake

The recommended intake for ω-3PUFAs is based on governing body. The Dietary Guidelines for Americans recommend consuming about 230 g/week of fish, corresponding to approximately 250 mg/day of EPA and DHA [29]. This recommended intake corresponds to consuming fish twice weekly, including one serving of oily fish. The U.S. Food and Drug Administration claimed that levels up to 3 g/day are considered as safe, while other authorities suggested at up to 5–6 g/day [79].

However, in intervention studies reporting a favorable health effect, the intake of fish oils or their derivatives resulted in long chain ω-3PUFAs daily intakes well above those “suggested” 200 mg/day and ranged from 0.5 to 9 g/day. Consequently, this justifies readjustments of nutritional guidelines to an upper level. Governments (UK, Belgium, The Netherlands, France, New Zeeland, and Australia) and health organizations (American Heart Association, FAO/WHO, American Dietetic Association,) now advise dietary consumption for total ω-3 PUFA of 1.4 to 2.5 g/day, with EPA and DHA ranging from 140 to 600 mg/day depending on the authority issuing guidelines, FOA/WHO making a relatively low recommendation of 250 mg/day, the average being around 500 mg/day [80]. This means minimum of 2 intake of fish/week (30–40 g/day), including one of oily fish (tuna salmon, sardine, and mackerel). In the light of the literature and inter individual changeability in PUFA metabolism and requirement, perhaps the minimal EPA+DHA supplies for healthy adults should reach 0.5–1 g/day (2–4 servings per week of fish, half of oily fish); that is minimal intake proved to reduce obesity and, in general, metabolic syndrome [81], with a total serving of ω-3 PUFA of 5–6 g/day as found in ancestral nutrition to which our metabolism is best fit [82]. Such levels are met in the traditional Japanese diet as it contains 80–100 g fish and shellfish/d/capita [81,83].

Numerous studies have discussed the actions of ω-3 PUFAs integration on obesity, in both animal and human models, highlighting possible mechanisms for ω-3PUFAs in decreasing body weight, improving body composition and counteracting the contrary metabolic effects of obesity [28,84]. However, clearly, findings of prospective studies concerning the favorable actions of ω-3 intake on obesity are far from agreement. The manifest discrepancies may have arisen due to differing or inadequate methods of data collection on food intake (food frequency questionnaires), changes in cooking procedures and other unaccounted for lifestyle behaviors (exercise, etc.) from study to study and among diverse study populations [29]. Therefore it is not possible at present to decide the ω3- dose to hinder obesity, neither through food, nor through integration. The advice is to act through nutritional interventions. In general, it is recommendable to replace the intake of SFAs with PUFAs. The World Health Organization (WHO) prompts eating at least two servings of oily fish per week, which is rich in the ω-3PUFAs (DHA and EPA) [85]. International and national guidelines on healthy eating agree in advising the intake of ω-3, both marine and vegetable, with a diverse and balanced diet, containing foods in which they are naturally present. In subjects at cardiovascular risk and on a diet low in these fatty acids, or in patients in secondary prevention, integration at diverse levels should be estimated with the specialized doctor [81]. Human intervention trials indicate potential benefits of ω-3PUFAs supplementation, especially when combined with energy-restricted diets or exercise, but more well-controlled and long-term trials are needed to confirm these effects and identify doses for antiobesity-action.

ω-6PUFAs are pro-inflammatory and commonly occur in poultry, eggs, corn, and most vegetable oils, and also in processed and fast foods. ω-6PUFAs have pro-inflammatory capability and are considered to be the counterpart of ω-3PUFAs, which are anti-inflammatory [18]. A high-fat diet and the Western dietary pattern feature high quantities of ω-6PUFAs and low amounts of ω-3PUFAs. Instead, a low-fat diet (e.g., traditional Japanese diet) is low in ω-6PUFAs and high in ω-3PUFAs [86]. Data show that a higher ratio of ω-6/ω-3PUFAs increases inflammation and the probability of chronic inflammatory syndromes, including cardiovascular disease, obesity, and nonalcoholic fatty liver syndrome [18]. Preclinical studies show that ω-6 PUFAs have a tumor-enhancing effect. In a recent Japanese cohort study, incorporating 38,200 women, ω-6PUFA intake was positively associated with breast cancer risk [81,87].

Many data discussed the importance of preserving a low omega–6/omega–3 ratio for decreasing inflammation. Decreasing the ω-6/ω-3 ratio seems to reduce the inflammatory response to a high-fat meal [88]. A stable ω-6/ω-3 is one of the most significant dietary factors in the inhibition of obesity: a lower ω-6/ω-3 ratio should be reputed in the management of obesity [89]. A high omega-6 fatty acid intake and a high ω-6/ω-3 ratio are connected with weight gain in both animal and human investigations, whereas a high ω-3 FAs intake reductions the risk for weight gain [89].

Several sources of information recommend that human beings evolved on a diet that had a ratio of ω-6 to ω-3PUFA of about 1/1; whereas today, Western diets have a ratio of 10/1 to 20–25/1, demonstrating that Western diets are lacking in ω-3PUFA related with the diet on which humans evolved and their genetic patterns were established [90,91].

Due to agribusiness and modern agriculture western diets enclose unnecessary levels of omega-6 PUFAs but very low levels of ω-3PUFAs, leading to an unhealthy ω-6/ω-3 proportion, instead of 1:1 that was during evolution [90]. It is thought that hominids’ foods during the Paleolithic era were high in seafood and low in seeds and vegetable oils, which led to a ω-6/ω-3 proportion of about 1:1 [30,92,93]. ω-6PUFAs are related to the synthesis of pro-inflammatory mediators while omega-3 PUFAs produce less powerful inflammatory mediators and inflammatory resolving proteins, so manipulating this proportion may bring about helpful health outcomes.

A balanced ω-6/ω-3 FA ratio (1:1 to 2:1 is optimal) is vital for homeostasis and regular development throughout the lifespan [92,94,95]. However, there are significant genetic variables in fatty acid biosynthesis including desaturase 1 and desaturase 2, which encode rate-limiting enzymes for FA metabolism. Data connected to genotyping of the desaturase region analyzed in human populations show that present-day humans vary dramatically in their capability to produce long-chain PUFAs [89,96].

In clinical investigations and intervention trials it is indispensable that the background diet is precisely determined in terms of the ω-6 and ω-3 FAs content. Because the concluding concentrations of ω-6 and ω-3PUFAs are defined by both dietary intake and endogenous metabolism, it is important that in all clinical investigations and intervention trials the ω-6 and ω-3 FAs are precisely defined in the red blood cell membrane phospholipids [89].

Mice fed the lowest ω-6/ω-3 ratio had the lowest non-HDL (i.e., atherogenic lipoporteins) and inflammation (IL-6). Mice fed lower ω-6/ω-3 ratio diets also had less macrophage cholesterol increase and less aortic atherosclerotic lesions. The lowest ω-6/ω-3 ratio (1:1) diet led to the least atherosclerotic formation and the severity of atherosclerosis augmented as the ω-6/ω-3 proportion increased [97].

Using long-chain ω-3PUFAs to suppress low-grade inflammation may advantage numerous chronic syndromes such as atherosclerosis, rheumatoid arthritis, diabetes, dyslipidaemia, obesity and heart failure. The ingesting of ω-6 seed oils may have the contrary action [98].

The consequences of extreme ω-6PUFAs remain controversial: ω-6PUFAs have intrinsic cardiovascular protective actions, justifying the latest FAO/WHO recommendations on maintaining high ω-6PUFAs consumptions if ω-3PUFA ones are fulfilled [99]. However, ω-6PUFAs compete with ω-3PUFAs for processing to eicosanoids, thereby limiting synthesis of anti-inflammatory ω-3PUFA derived mediators [100]. Moreover, there are convincing proofs that a low ω-6/ω-3PUFA ratio is determinant for the inhibition of pathologies connected to the metabolic syndrome, as colorectal cancer [101].

Deduced from ancestral nutrition, in an ideal balanced diet, fat should represent no more than 20–30% of total energy intake amongst which 5–6 g/day of ω-3PUFAs with a great percentage of EPA+DHA and the ω-6-to-ω-3 proportion should average 1 [102,103]. To keep in with a developmental approach and with the epigenetic consequences of the diet, a proportion of ω-6/ω-3 around 1 in breast milk should serve as a bench mark to decide the correct dietary requirements during pregnancy, lactation, and infant feeding [81,104].

## 4. Role of ω-3PUFAs in Inflammation and Colorectal Cancer Development

A large body of literature highlights the importance of dietary intake for the risk and progression of chronic disorders including inflammatory and neoplastic disease [105]. Nowadays it is well known that inflammation is a predisposing factor for cancer capable to promote the insurgence of several types of tumors [106], and that an inflammatory microenvironment is an essential component of all tumors. During metastasis development it has been shown that microenvironment modulates the capability of tumor cells and cancer stem cells to evade the innate immune response and survive. The metastatic niche is a complex system including several cell types, as vascular, stromal, and above all inflammatory and immune cells, in addition to many other molecules which provide survival, immune surveillance protection and metabolic requirements. The interaction among all these factors determines metastatic dissemination [107].

Only a few of all cancers depend on germline mutations, while the majority is determined by somatic mutations and environmental factors. Most often cancer is caused by chronic inflammation such as chronic infections, tobacco smoke, inhalation pollutants (such as silica and asbestos), and dietary factors (some forms of cancer are linked to obesity) [108]. Furthermore, in some cases exogenous diet-derived miRNAs might substantially contribute to the pool of circulating miRNAs, regulating tissue homeostasis and interfering with human health [109].

CRC is a multifactorial disease caused by multiple genetic and environmental factors. These include the type of diet, the lifestyle, the intake of alcoholic beverages, smoking, obesity, genomic abnormalities, alterations in the signaling pathways, chronic activation of the inflammatory response, oxidative stress, dysbiosis, etc. These factors work by altering intestinal homeostasis [110]. In fact, despite still lacking extensive epidemiological studies to date, most cases of early-onset colorectal cancer (EO-CRC) arise sporadically and are attributable to environmental factors [111]. In recent years, due to the activation of preventive screening activated in the population aged ≥50 years, the incidence of CRC in Western countries has stabilized and even decreased. In contrast, the incidence of CRC among people under the age of 50 has increased in both Europe and the United States, thereby representing a major public health problem [112,113]. However, CRC remains the fourth leading cause of cancer death in the world dependent on a close relationship between inflammation and environmental factors [114].

The gastrointestinal tract is not only responsible for digestion and absorption of nutrients but also represents a powerful barrier against pathogens and toxins harmful to the individual. It also has an endocrine function responsible for maintaining the metabolic homeostasis of the whole organism. Since the intestine comes into direct contact with food, it is very sensitive to dietary factors which directly influence both its structure and function [115]. In fact, the crypts and intestinal villi are structurally influenced by external factors by changing their size in response to changes in the diet [116].

Since chronic inflammation has been shown to promote the onset of CRC in humans, ω-3PUFAs, due to their strong anti-inflammatory function, have been shown to be protective against colon cancer [117,118].

Some studies have been conducted on the efficacy of ω-3PUFAs in the prevention of CRC through the integration of purified EPA with DHA or fish oil (FO). These studies have shown the importance of ω-3PUFAs in inhibiting the uncontrolled proliferation of CRC cells both when administered in large quantities for short times (8–9 g of EPA+DHA/day for 2 weeks) and in smaller quantities for longer times (2.5–4 g of EPA+DHA/day for 3–6 months). However, the effect on the control of intestinal cell proliferation was not seen in patients with the same supplementation but with a high-fat basal diet and a low ω-3/ω-6PUFAs ratio. For this reason, the effectiveness of ω-3PUFAs depends on both the total lipid content and the ω-3/ω-6PUFAs ratio [119].

Many studies have been conducted in CRC models to explain the molecular mechanisms to the base of the anti-inflammatory and anti-neoplastic activity of ω-3PUFAs. First of all, ω-3PUFAs are incorporated in phospholipid membrane inducing an alteration in structure, fluidity and function of lipid rafts. These membrane changes influence the activity of membrane receptors leading at the inhibition of signaling pathways involved in the activation of pro-inflammatory molecules of cell survival and apoptosis [120,121,122]. Moreover, in CRC, ω-3PUFAs modulate inflammatory pathways, generating lipid mediators implicated in the resolution of inflammation including resolvine, protectin, and maresins [123].

The ω-3PUFAs exert their antitumor actions through different mechanisms, involving proliferation, apoptosis, and migration. Their affects involve COX-dependent or COX-independent mechanisms, and they act on different pathways such as Wnt/β-catenin and Hippo or by regulation of oxidative stress and the expression of Granzyme B. To date, there are numerous papers in the literature that describe different mechanisms of action of ω-3PUFAs in CRC, as summarized in Table 1.

Multiple molecular mechanisms causing an increased apoptosis of CRC cells depend on the action of ω-3PUFAs. First of all, ω-3PUFAs influence the redox state of the cells: indeed, there is a link between anti-tumor effects of ω-3PUFAs and oxidative stress. PUFAs may induce an increase in apoptotic potential of CRC cells by enhancing the concentration of intracellular reactive oxygen species (ROS), inducing an elevated cancer cells apoptosis by the loss of mitochondrial membrane potential, ROS generation, activation of caspase 3 and 9, and by an increase of Bax/Bcl2 ratio [124].

Another important anti-inflammatory mechanism involves COX, a major player in inflammation. COX hyperactivation in the CRC, induces in turn the production of PGE2, a powerful pro-inflammatory and pro-carcinogenic agent [125]. ω-3PUFAs exert their anti-inflammatory role by modulating COX activity. In this respect, EPA, acting as an alternative substrate for COX-2, induces a switch in production from pro-tumorigenic PGE2 to three series PGs (PGE3) that abrogate the antiapoptotic activity of PGE2 in CRC cells [126]. However, the anti-cancer mechanism of ω-3PUFAs in CRC could also be explained by a COX2-indipendent mechanism. Indeed, DHA and EPA inhibit the proliferation and induce the apoptosis of CRC cells in vitro and in animal models. At molecular level, involvement of the Hippo pathway, cytoplasmic retention of phosphorylated YAP by GPRs (GPR40 and GPR120)-Gαs-PKA cascade has been reported [127]. Moreover, the GPR120 is expressed on macrophages and regulates their polarization reducing inflammation [128].

In addition to apoptosis, ω-3PUFAs can also influence proliferation and migration capability of CRC cells. In colorectal cancer, the Wnt-β-catenin signaling pathway is the key regulator of tumor development, and alterations in this cellular signaling pathway can be found in most patients [129]. It has been shown that dietary ω-3PUFAs are able to inhibit significantly intestinal polyp growth in mice, correlating with the ECS described in Section 2.1. In fact, CB1 up-regulation reduces β-catenin and its transcriptional target c-myc, both involved in regulation of cell proliferation. In CRC patients, cancer tissue shows a significant inhibition of CB1 expression levels, compared to adjacent normal tissue, demonstrating that the “protective” action of endocannabinoids via CB1 is lost in the tumor [130]. Moreover, D’Eliseo and colleagues have studied the effect of DHA on migration of CRC cells and demonstrated that DHA inhibits Granzyme B expression, reducing CRC cells capacity to undergo epithelial mesenchimal transition (EMT) and invade matrigel [131].

Finally, ω-3PUFAs regulate the expressions of genes involved in inflammation and colon cancer development also through epigenetic modifications [132,133,134]. In fact, more recently, ω-3PUFAs have been attributed the ability to influence the epigenetic regulation of genes involved in the polarization of macrophages, negatively regulating the colorectal carcinogenesis; however, the interesting topic is not fully understood [135].

Taken together, although the many different mechanisms, ω-3PUFAs play anti-inflammatory and anticancer effects acting on the classic hallmarks of cancer, i.e., cell proliferation, apoptosis and migration.

To date, one of the most important problems in the treatment of tumors, including sporadic colorectal cancer, is the development of resistance to anti-tumor treatments and tumor relapse that can be related to self-renewing of cancer stem/stem-like cells (CSC/CSLC) within a tumor mass. Many groups have studied the effects exerted by ω-3PUFAs on cancer stem-like cells.

With the immunophenotyping of CSLC, the anti-CD133 antibody was found to be effective for isolating a population of colon cancer cells that retained the properties of stem cells (CSLC), while anti-cytokeratin 20 (CK20) and anti-Mucin-2 (MUC2) were specific epithelium colonic differenziation markers. EPA treatment induces an increase in of CK2 and MUC2 and an inhibition of CD133 expression. This means that the EPA could induce a more differentiated state of most cancer cells and could trigger the reduction of the stem state of the CSLC, as demonstrated by the reduction of the expression of the CD133 marker [136].

Moreover, Yang and colleagues showed an antiproliferative and proapoptotic effect on the dedifferentiated SW620 colon cell line, treated with DHA and EPA [137].

Additionally Sam et al., evaluating the effects of DHA and EPA treatments on LS174T cells, a model for colorectal cancer initiating cells with stem cell-like properties, demonstrated that ω-3PUFAs induce cell growth inhibition and promote cell death by down-regulating survivin expression and activating caspase-3 [138].

The effect of ω-3PUFAs on CSLC may be an important goal for cancer therapy and will constitute an interesting challenge for future studies. Anyway, the anti-tumor activity of ω-3PUFAs, shown through multiple mechanisms, suggests that they could have an important therapeutic role in the management of CRC.

## 5. Conclusions

Obesity is a preventable disease that can be treated through proper diet and exercise. A balanced ω-6/ω-3 ratio 1–2/1 is an important dietary factor in the prevention of obesity, along with physical activity. Different pro- and anti-inflammatory properties are exerted by ω-6 and ω-3PUFAs themselves and by their derivatives, such as endocannabinoids, lipid mediators deeply involved in the control of many biological functions, including the inflammatory response and the central and local control of food intake and energy homeostasis. Therefore, appropriate dietary intervention has primarily relevance in the prevention and the treatment of obesity in that it maintains the efficiency of key signaling pathways and avoids long term/chronic inflammatory states.

Inflammation is a predisposing factor for cancer, CRC included, with ω-3PUFAs exhibiting anti-cancer properties, once again confirming the need for a balanced ω-6/ω-3 ratio for health preservation.

The discovery of cancer stem cells offers a new perspective in cancer therapy. Since CSCs contribute to cancer onset and relapse after conventional therapy, they can represent a unique fundamental therapeutic target to completely cure cancer. Thus, the effect of ω-3s on CSLC may be an important goal for cancer therapy and will constitute an interesting challenge for future studies. Anyway, the anti-tumor activity of ω-3s, performed through multiple mechanisms, suggests that they could have an important therapeutic role in the management of CRC.

## Figures and Tables

**Table 1:** ω-3PUFAs target different molecular pathways acting on classical hallmarks of cancer, i.e., proliferation, apoptosis, and migration.

Mechanism of Action	Effects	References
Regulation of oxidative stress	Activation of apoptosis	[124]
Regulation of COX metabolism	Activation of apoptosis	[125,126]
Regulation of Hippo pathway via GPRs-Gαs-PKA cascade	Activation of apoptosis, cell proliferation inhibition	[127,128]
Wnt/β-catenin pathway regulation	Cell proliferation inhibition	[129,130]
Inhibition of Granzyme B expression	Inhibition of EMT, Inhibition of migration	[131]

Abbreviations: COX: cyclooxygenase, GPR: G-coupled receptor, Gαs: G-protein alpha subunit, PKA: protein kinase A, EMT: epithelial mesenchymal transition.

## Abbreviations

AA	arachidonic acid
AEA	anandamide
2-AG	2-arachinonoylglycerol
AgRP	agouti related protein
ALA	α-linolenic acid
AMPK	AMP-activated protein kinase
ARC	hypothalamic arcuate nucleus
BAT	brown adipose tissue
BMI	body mass index
CART	cocaine-amphetamine regulated transcript
CB	cannabinoid receptor
CK	cytokeratin
COX	cyclooxygenase
CRC	colorectal cancer
CSC	cancer stem cell
CSLC	cancer stem-like cell
DHA	docosahexaenoic acid
DHEA	N-docosahexaenoyl ethanolamine
DPA	decosapentaenoic acid
ECS	endocannabinois system
EMT	epithelial mesenchymal transition
EO-CRC	early-onset colorectal cancer
EPA	eicosapentaenoic acid
EPEA	N-eicosapentanoyl ethanolamine
EPG	eicosapentaenoyl glycerol
FA	fatty acid
FFAR	free fatty acid receptor
GPR	G-coupled receptor
HFD	high fat diet
IL-	Interleukin-
LA	linoleic acid
LPS	lipopolysaccharide
MCH	melanin-concentrating hormone
MCP-1	monocyte chemo attractant protein 1
MUC	mucin
NPY	neuropeptides like neuropeptide Y
PAI-1	plasminogen activator inhibitor
PGE2	prostaglandin E2
POMC	proopiomelanocortin
PPAR	peroxisome proliferator-activated receptor
PUFA	polyunsaturated fatty acid
RBC	red blood cells
ROS	reactive oxygen species
SFA	saturated fatty acid
TNF-α	tumor necrosis factor-alpha
TRPV1	transient receptor potential cation channel subfamily V member 1
UCP1	uncoupling protein 1
WAT	white adipose tissue
